# Transient Dip in Head Circumference Growth Trajectories Associated With Continuous Positive Airway Pressure Administration in Very Preterm Infants

**DOI:** 10.1111/apa.70392

**Published:** 2025-12-05

**Authors:** Elena‐Laura Dumitrescu, Christoph Bührer

**Affiliations:** ^1^ Department of Neonatology Charité Universitätsmedizin Berlin Berlin Germany

**Keywords:** continuous positive airway pressure, head circumference, noninvasive respiratory support, trajectories, very preterm infants

## Abstract

**Aim:**

In preterm infants, decreasing head circumference (HC) percentiles have been linked to neurodevelopmental impairment. Devices for administering continuous positive airway pressure (CPAP) may alter skull morphology. We investigated the impact of sequentially administered CPAP and high‐flow nasal cannula on HC percentiles.

**Methods:**

In this retrospective single‐institution study of infants < 1500 g birthweight and < 30 weeks gestational age born 2019–2020 (syndromes and intraventricular haemorrhage ≥ grade 2 excluded), routine HC measurements were translated into HC percentiles. We compared data at birth, after CPAP initiation, after partial and complete discontinuation of CPAP, and at discharge.

**Results:**

The study included 137 infants (median [interquartile range] gestational age 27^+3^ [25^+5^–28^+6^] weeks, birthweight 890 [715–1120] g). After initiation of CPAP, HC percentiles dropped from 57 [18–77] to 16 [2–26] (*p* < 0.001), and declined further until CPAP was partially replaced by high‐flow nasal cannula to 7 [3–30] (*p* < 0.001). HC percentiles increased after total weaning off CPAP to 14 [3–30] (*p* = 0.004) and continued to rise until discharge to 22 [8–37] (*p* < 0.001).

**Conclusion:**

CPAP was associated with a transient decline of HC percentiles, with gradual recovery after CPAP discontinuation.

## Summary

- At birth, skull sutures are partly open to allow for adaptation of the infant's head to external forces.
- In very preterm infants, prolonged administration of continuous positive airway pressure (CPAP) was associated with a transient decline of occipitofrontal head circumference percentiles and *z* scores.
- Head circumference percentiles and *z* scores showed gradual recovery after CPAP discontinuation.

**Abbreviations**
CPAP: continuous positive airway pressure
HC: head circumference
HFNC: high‐flow nasal cannula
IQR: interquartile range

## 1 Background

Two cohort studies reported an inverse relationship between the average rate of weekly in‐hospital head circumference (HC) growth and neurodevelopmental scores at 18–22 months [1, 2]. However, little is known about how clinical practice might shape head growth. Infants randomised to receive more amino acids and calories during the first weeks of life were found to have higher HC *z* scores as opposed to those in the standard group [3]. This did not translate into better motor, language, or combined neurodevelopmental scores at 2–3.5 years of age [4]. Subsequent attempts to promote head growth in hospitalised preterm infants by a variety of nutritional interventions have largely failed. This includes intensified nutrition with parenteral amino acids [5], enteral protein [6], polyunsaturated fatty acids [7], or iron [8]. In Germany, the relative loss of HC for postmenstrual age percentiles between admission and discharge has been viewed as an indicator of quality of care for preterm infants [IQTIG 2023].

At birth, skull sutures are partly open to allow for adaptation of the infant's head to external forces. Administration of continuous positive airway pressure (CPAP) requires devices attached to the infant's head. Inadvertently, these devices exert some pressure. A pilot study has shown that various CPAP devices may gear the head shape towards a turricephalic deformation [9]. Cumulative exposure time to caps for CPAP administration has been reported to be negatively associated with HC and ear‐to‐ear distance *z* scores [10]. We hypothesized that HC growth trajectories are influenced by the type of noninvasive respiratory support administered. The present study investigates the impact of CPAP, followed by high‐flow nasal cannula (HFNC), on routinely obtained occipitofrontal HC measurements in very preterm infants between birth and discharge.

## 2 Methods

In this single‐institution retrospective analysis, anonymized routine weight and HC measurements of preterm infants below 1500 g birth weight and less than 30 weeks gestational age born 2019–2020 and admitted within 24 h after birth were retrieved from electronic charts. Infants with recognised syndromes, intraventricular haemorrhage ≥ grade 2, or incomplete data were excluded.

CPAP was administered interchangeably via the Fisher‐Paykel flexitrunk interface (Fisher & Paykel Healthcare Limited, Auckland, New Zealand) or the EasyFlow nCPAP accessory (Fritz Stephan GmbH, Gackenbach, Germany). Both devices consist of nasal prongs or a nasal mask, bonnet, and headgear secured to the infant's head with straps and clips.

Weight and head circumference were considered at 5 time points: first measurement after birth, after definitive start of binasal CPAP with prongs or mask, after start alternation between CPAP and HFNC, after definitive end of CPAP, and the last measurement prior to discharge.

Raw data were transposed into gestational age‐specific and sex‐specific z‐scores and percentiles based on the Fenton growth charts [11] via the Kinderarztrechner online calculator [12]. For infants below 23 weeks gestational age, data from the German perinatal survey were used [13]. SPSS 30.0 (IBM, Armonk, N.Y., USA) was used for the statistical analysis. Data were compared using the related‐samples Friedman analysis of variance followed by paired Wilcoxon tests for adjacent time points, with 2‐tailed *p* values < 0.05 considered to indicate statistical significance.

All procedures were performed in compliance with relevant laws, institutional guidelines, and the Declaration of Helsinki, as revised in 2024. The study received approval from the local institutional review board (Ethikkommission Charité—Universitätsmedizin Berlin, number EA2/199/22) which waived the need for informed parental consent.

## 3 Results

Between 1 January 2019 and 31 December 2020, a total of 224 infants below 1500 g birth weight and a gestational age below 30 weeks were admitted. Of these, 33 had intraventricular haemorrhage grade 2 or more, 43 died, and 10 had incomplete data for other reasons. The final study cohort comprised 137 infants (64 girls, 73 boys) with a median gestational age of 27^+3^ weeks^+days^ [interquartile range (IQR) 25^+5^–28^+6^] and a birth weight of 890 [715–1120] g.

Table 1 presents the chronological and postmenstrual age at which measurements were taken after birth, after definitive start of binasal CPAP, after start alternation between CPAP and HFNC, after definitive end of CPAP, and the last measurement prior to discharge.

**TABLE 1:** Postmenstrual age and chronological age [median, interquartile range] at birth, start of binasal CPAP, start alternation between CPAP and HFNC, definitive end of CPAP, and prior to discharge.

	Birth	CPAP	CPAP/HFNC	End CPAP	Discharge
Postmenstrual age (weeks^+days^)	27^+3^ 25^+5^–28^+6^	29^+2^ 28^+0^–29^+2^	31^+6^ 31^+0^–31^+6^	33^+5^ 32^+2^–35^+0^	37^+0^ 36^+0^–39^+1^
Chronological age (days)	0 0–0	9 6–13	27 17–49	46 26–65	70 53–90

Head circumference percentiles continuously declined from birth during the administration of CPAP, then rose steadily after partial and definitive withdrawal of CPAP until discharge (Table 2).

**TABLE 2:** Head circumference percentiles [median, interquartile range] at birth, after start of CPAP, start alternation between CPAP and HFNC, definitive end of CPAP, and prior to discharge.

Birth	*p*	CPAP	*p*	CPAP/HFNC	*p*	End CPAP	*p*	Discharge
57	< 0.001	16	< 0.001	7	< 0.001	14	< 0.001	22
18–77		4–38		2–26		3–30		8–37

**FIGURE 1:**
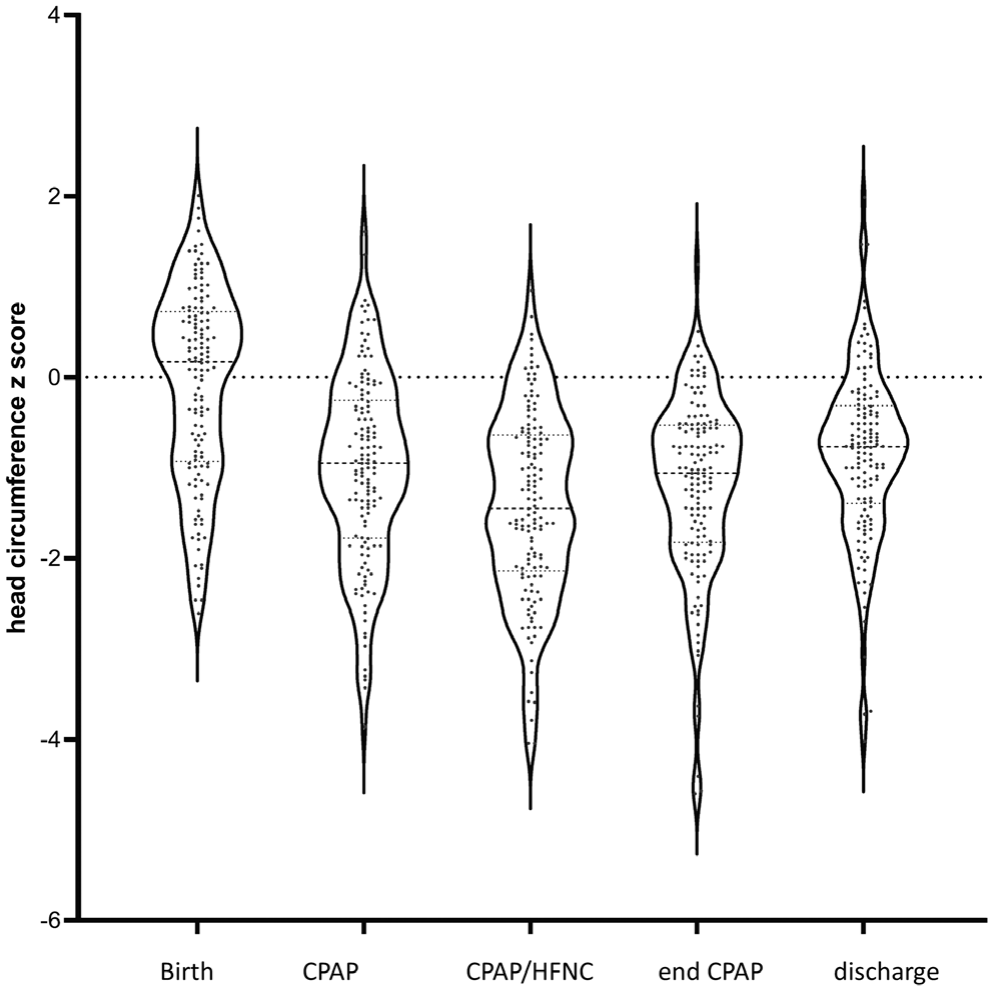
Violin plots of head circumference *z* scores at birth, after the start of CPAP, start of alternation between CPAP and HFNC, definitive end of CPAP, and prior to discharge.

A similar picture emerged when head circumferences were expressed in *z*‐scores (Figure 1).

The ratios of head circumference percentile to body weight percentile also declined during CPAP and recovered until discharge (Table 3).

**TABLE 3:** Ratios of head circumference percentile to body weight percentile [median, interquartile range] at birth, after the start of CPAP, start alternation between CPAP and HFNC, definitive end of CPAP, and prior to discharge.

Birth	*p*	CPAP	*p*	CPAP/HFNC	*p*	End CPAP	*p*	Discharge
1.15	< 0.001	0.81	< 0.001	0.61	< 0.001	1.00	< 0.001	1.87
0.80–1.69		0.36–1.29		0.23–1.17		0.61–1.81		1.06–4.00

## 4 Discussion

In this cohort of very preterm infants, occipitofrontal HC deviated downwards from intrauterine trajectories while infants were treated with CPAP. This was followed by a catch‐up increase upon CPAP discontinuation. This was not mediated by postnatal growth failure, as the ratios of head percentile to body weight percentile declined during CPAP but fully recovered by discharge, exceeding values at birth.

While previous studies have looked at HC at birth and at discharge, this is the first study examining HC during the entire course of the hospital stay in relation to the type of respiratory support administered. This strength is offset by three limitations. First, the study relied on routine measurements which included occipitofrontal HC but not ear‐to‐ear distances or other measurements. Therefore, we were unable to decide whether the reduced HC in the wake of CPAP represented smaller head volumes or was compensated for by eccentric head growth. Second, the two CPAP devices had been used interchangeably during the study period. Therefore, we could not determine any difference between the two of them. Third, CPAP having become standard of care for preterm infants < 30 weeks gestational age, there was no control group without CPAP.

CPAP‐related transient decline in head circumference for postmenstrual age percentiles may underlie the apparent nadir reported for head circumference between birth and follow‐up measurements at 3–12 months of age [14, 15]. Infants with poor head growth between birth and discharge followed by catch‐up post discharge were found to have a reduced odds ratio of significant neurodevelopmental impairment [16].

In more recent cohorts of preterm infants, the association between changes in HC *z* scores between birth and discharge and neurodevelopment outcome has faded. In extremely preterm infants below 29 weeks gestational age cared for in hospitals of the Canadian Neonatal Network, changes in HC between birth and discharge were neither related to Bayley‐III scores at 18–24 months corrected age nor to neurodevelopmental impairment [17]. In contrast, there were some small associations between head growth post‐discharge and neuromotor development. In a single‐institution study in Leipzig, Germany, no correlations emerged between cognitive abilities at 2 years and the slopes of HC between birth and discharge [10]. A population‐based cohort study including all live‐born infants below 28 weeks gestational age in 2006–2012 in Switzerland also found no association between declining head circumference *z*‐scores between birth and discharge and neurodevelopmental impairment at 2 years of age [18].

## 5 Conclusion

Data of this study suggests that the HC trajectories in the hospitalised very preterm infants studied were influenced by devices to deliver non‐invasive respiratory support. Apparent loss of HC percentiles during CPAP was reversed upon discontinuation of CPAP. These observations may explain the poor predictive power of HC at discharge for future neurodevelopment.

## Author Contributions

**Elena‐Laura Dumitrescu:** project administration, investigation, data curation, writing – review and editing, validation. **Christoph Bührer:** conceptualization, methodology, formal analysis, supervision, writing – original draft.

## Funding

The authors have nothing to report.

## Conflicts of Interest

The authors declare no conflicts of interest.

## Data Availability

The data that support the findings of this study are available on reasonable request from the corresponding author after approval of the Ethikkommission and Data Protection Officer of the Charité – Universitätsmedizin Berlin.
